# Editorial: Improvement for Quality and Safety Traits in Horticultural Plants

**DOI:** 10.3389/fpls.2022.927779

**Published:** 2022-05-31

**Authors:** Xinyang Wu, Pingping Fang, Peipei Zhang, Ting Sun, Xinchao Wang, Ferdinando Branca, Pei Xu

**Affiliations:** ^1^College of Life Sciences, China Jiliang University, Hangzhou, China; ^2^Key Laboratory of Specialty Agri-Product Quality and Hazard Controlling Technology of Zhejiang, Hangzhou, China; ^3^National Center for Tea Improvement, Tea Research Institute, Chinese Academy of Agricultural Sciences, Hangzhou, China; ^4^Key Laboratory of Biology, Genetics and Breeding of Special Economic Animals and Plants, Ministry of Agriculture and Rural Affairs, Hangzhou, China; ^5^Departments of Agriculture, Food, and Environment, University of Catania, Catania, Italy

**Keywords:** quality, safety, horticultural plants, phenotyping, genetics, integrative biology quality

As the living standard of people rises, quality and safety traits of agricultural plants have received growing attentions by consumers and breeders alike. Quality traits are known as traits that offer better appearance, flavor, taste, nutrition, longer shelf life and less damage to plant products, while safety traits can be defined as plant traits that are directly or indirectly linked to human health, such as the content of antioxidant compounds in horticultural products. Typical quality and safety traits include, but are not limited to, appearance, texture, taste, flavor and aroma of plant products; nutritional, anti-nutritional or allergic component contents; uptake, accumulation and degradation of exogenous toxic substances by plants; synthesis and transformation of endogenous harmful substances in plants; post-harvest quality change and shelf life. Some quality and safety traits are more closely related to the environment or human activities, such as the accumulation of heavy metals, antibiotic residues and the novel artificial nanoparticles in plants. Tremendous efforts are being put into breeding programs to synergistically improve quality and safety traits, which, however, are in some cases impeded by the trade-offs among target traits. For example, plants may contain certain components, for instance, stone cells, anti-nutritional factors and allergens, which are beneficial to their own but unfavorable for human consumption or detrimental to health (Aranzana et al., 2019; Lin et al., 2022).

## Quality and Safety Traits Are Receiving More Attentions in Horticultural Plants

Horticultural crops generally refer to fruits, vegetables, floral and tea plants. Fresh fruits and vegetables are important parts of human diets because they are essential sources of nutrients (e.g. carbohydrates, minerals, vitamins) and dietary fiber. Tea plant has huge health benefits and economic values. Tea contains many secondary metabolites such as flavonoids, theanine and alkaloids, which play many health benefits in human body (Yang et al., 2009; Bag et al., 2021). Floral plants, by their decorative function and cultural value, are an indispensable component for quality life and gaining more importance in modern agriculture. Collectively, horticultural crops are playing versatile roles in meeting the diversified needs of people and the rapidly upgrading life quality. As such, compared to staple food crops, for which yield-related traits are more critical, quality and safety traits are of particular importance for horticultural plants. Driven by the increasing demand of high-end horticultural products, quality and safety traits are receiving unprecedently intense attentions around the globe and have become a research hotpot. This Research Topic presents 24 articles that report the recent progresses in various subjects of research on quality and safety traits in vegetable, fruit, beverage and floral plants. In this editorial article, we briefly summarize the recent progresses and provide prospective views of the future directions in this specific area.

## Progress in Phenotyping of Quality and Safety Traits in Horticultural Plants

Accurate acquisition of the phenotypic data is prerequisite for trait improvement. The past decades have witnessed big progress in phenotyping techniques and methods, covering a wide range of traits from morphological, biochemical to the physiological levels. For example, advanced mathematical algorithm such as Elliptic Fourier equation was employed in precise and comprehensive quantification of fruit shape in cucurbit crops (Xu et al., 2021). Recently, the Tomato Analyzer image toolkit provides quantitative and objective description for morphological traits of fruits, which are fundamental for the characterization, selection and breeding of cultivars with desired fruit appearances (Brewer et al., 2006; Hurtado et al., 2013). Similarly, Zhu et al. developed an automated method for measuring color and size indicators of tomato fruits by incorporating a deep learning model. Various phenotypes such as fruit color, horizontal and vertical diameters, top and navel angles, locule number, pericarp thickness can be extracted, providing an effective and accurate means of fruit phenotyping. The same research group also developed a method for measurement of soluble solids content (SSC) and fruit firmness based on hyperspectral images and deep learning regression, which offers a new solution for non-destructive assessment of cherry tomato fruit quality (Xiang et al.).

Phenomics, an emerging discipline that provides high-throughput quantification of traits, has exhibited unique advantages in characterizing traits of horticultural plants (Li et al., 2021; Pandey et al., 2021). Current phenomics techniques are able to capture the dynamic process of plant growth and responses to the environment. For example, non-destructive plant imaging combined with destructive leaf sampling allowed deep analysis of salt resistance (Berger et al., 2012). By employing non-destructive physiology-based phenomics assay, drought responses such as stomatal closure of tomato, cowpea and pepper were precisely and continuously phenotyped (Berger et al., 2010; Xu et al., 2015; Halperin et al., 2017; Dalal et al., 2019; Wu et al., 2021). Even though the phenomics characterization of quality and safety traits in horticultural crops has still been rare to date, a wave of phenomics-based technological revolution on phenotyping of these traits is expected to come soon in the near future.

## Progress in Genetic Studies of Quality and Safety Traits in Horticultural Plants

As the genomic technologies burst in the recent decades, genetic dissection of a large number of quality and safety traits have become feasible in many horticultural plants, which solidifies the basis of genetic improvement of the traits. Particularly, pedigree-based genetic mapping using bi-parental populations such as second filial generation (F_2_), recombinant inbred lines (RILs) or double haploids (DHs); genome-wide association studies (GWAS) based on natural populations; and faster approaches using bulked-segregant analysis sequencing (BSA-seq) or quantitative trait locus sequencing (QTL-seq) have been mostly applied. For example, a locus strongly associated with firmness and harvest date of fruit was identified in apple by GWAS and the causal gene was found to encode the transcription factor *NAC18.1*. Single nucleotide polymorphisms (SNPs) were found in both the CDS and promoter regions of *NAC18.1*, leading to the differential ripening programs among the apple accessions (Migicovsky et al., 2021). Another interesting example reported the identification of a non-bitter allele fixed in *C. lanatus* by genome resequencing, which helped understand the domestication history of watermelons and their improvement (Guo et al., 2019). Shen et al. identified the melon gene *MELO3C026282* involved in the development of chloroplast to be a candidate gene controlling the mottled rind trait. Another gene, *MELO3C019694*, which encodes a MADS-box transcription factor, was characterized as a recessive candidate gene governing melon fruit surface groove by Du et al. using GWAS and BSA-seq. By genetic analysis of F_2_ and F_2:3_ populations derived from the cross of “MR-1” (green stigma) and “M4-7” (yellow stigma), Lv et al. detected two stable QTLs (SC2.1 and SC8.1) related to stigma color. Apart from melon, the formation mechanism and determinants of fruit quality in cucumber was reviewed by Zhang et al. highlighting the importance of integrating the methods of traditional breeding, molecular markers and bio-technologies. Other than quality traits, studies on diseases resistances of cucumber were summarized by Miao et al., where recent discoveries on the inheritance, molecular markers and QTL mapping of various diseases were reviewed, which sheds lights on the strategy for resistance breeding in this crop.

The completeness of genome sequencing in many horticultural plants have allowed genome-wide identification of genes involve in quality and safety traits. Zhu et al. identify 34 *N6-methyladenosine* regulatory genes and Li et al. characterize 51 *pectin methylesterase inhibitor* (*PMEI*) genes in tea. In pepper, 158 *carotenoid cleavage oxygenase* (*CCO*) genes were identified by Yao et al. and 10 *CONSTANS-like* (*COL*) genes were characteized by Huang et al. and Wang et al. reported the identification of 32 *terpene synthases* (*TPS*) genes in *Cymbidium faberi*. In short, the elucidation of genetic architectures of many important quality and safety traits and the systemic identification of gene families responsible for the trait regulation have laid a foundation for breeding-by-molecular-design.

## Integrative Biology Approaches for the Study of Quality and Safety Traits in Horticultural Plants

Integrative biology features the integration and intersection of multiple disciplines to study the laws of living organisms at the systems level (Pazhamala et al., 2021). In particular, genomics, phenomics, transcriptomics and proteomics, along with state-of-the-art gene manipulation and synthetic biology technologies, have driven the studies of quality and safety traits into the systematic era.

A typical example of integrative biological study of quality and safety traits in horticultural plants is the dissection and manipulation of the bitterness trait in cucumber. Through large-scale phenotyping and genetic mapping, genomic regions governing fruit and leaf bitterness were delimited. Further facilitated by biochemical and molecular approaches, nine genes involved in the cucurbitacin C biosynthesis pathway were identified, and two transcription factors, Bl and Bt, were ultimately determined as the causal gene of leaf and fruit bitterness, respectively (Shang et al., 2014). Through a comparative genomic approach, Zhou et al. unveiled the conserved mechanism and syntenic loci governing bitterness trait in the related cucurbit species including melon and watermelon. Based on genetic and metabolic analyses, it is revealed that the phytochemicals (phenolics, ascorbic acid, glucosinolate, anthocyanin, carotenoids) have great diversity among populations of Brassica wild species (Branca et al., 2018; Picchi et al., 2020). Ren et al. elucidated the mechanisms underlying selenium absorption and assimilation in tea by a combinatory use of transcriptomic and proteomic approaches. They found that the predominant forms of bioavailable Se were selenite (SeO${}_{3}^{2-}$) and selenate (SeO${}_{4}^{2-}$). Certain phosphate transporter (*PHT3;1a, PHT1;3b*, and *PHT1;8*) and aquaporin (*NIP2;1*) genes were upregulated by selenite while transporter genes *SULTR1;1* and *SULTR2;1* were responsive to selenate. Using tissue culture and transcriptomic profiling, Chen et al. showed that activated carbon promoted the development of peony roots into seedlings by stimulating the processes of phenylpropanoid biosynthesis and biosynthesis of stratum cutin and suberin. In addition to omics, research paradigm integrating physiological, biochemical and molecular biological methods are also widely used. For example, exogenous application of 5-Aminolevulinic acid and melatonin enhanced the nutritional quality and changed mature period in tomato (Wang et al.) and baby mustard (Di et al.), respectively. Zhu et al. employed high-performance liquid chromatography (HPLC) analysis, comparative transcriptome sequencing and transient expression assay to investigate the regulation of floral pigmentation in orchids. *CkCHS-1, CkDFR*, and *CkANS* were identified as putative key genes regulating floral coloration. Another study by Wen et al. analyzed CcERF2, an ethylene-inducible transcription factor that preferentially expressed in the placenta of pepper fruit. Silencing *CcERF2* reduced the content of capsaicinoids in pepper, suggesting that the gene involved in the regulation of capsaicinoids biosynthetic pathway. In a review article, Wang et al. summarized the recent research on the evolution and biological functions of the S1-bZIP transcription factors in plants, highlighting their role as signaling hubs through dimerization with cofactors to coordinate the expression of downstream genes. Undoubtedly, integrative biology approaches have been critical for understanding how the many quality and safety traits are determined in horticultural plants.

## Outlook

Despite a rapid advancement in the studies of quality and safety traits in horticultural plants over the past decades, improvement of these traits has still been lagging behind other essential traits, especially yield- and stress-related traits. Two main reasons are considered to be underpinning this fact: (1) phenotyping of quality and safety traits is generally more difficult; and (2) many of these traits are governed by multiple genes that are more prone to be affected by the environment. In order to accelerate the improvement of these traits, the research community may need to first fully digest and utilize the recent fundamental discoveries in the biology of quality and safety traits in horticultural plants. Particular attentions may be paid to latest research progresses in the innovative phenotyping techniques, exploration of genetic variations, important genes/QTLs that were identified as controlling the interested traits, molecular markers that were developed to tag the target traits, and the gene regulatory mechanisms underlying traits formation. In addition, Gao et al. emphasized the necessity and importance of establishing efficient genetic transformation methods and the use of gene-editing technology. These biotechnologies will help verify the functions of the determinant genes/QTLs.

In summary, the series of researches here describe the latest progresses on quality and safety traits studies in horticultural crops and highlight the combination of phenotyping and genetic methods in this field. In the future, researches on plant quality and safety traits will benefit more from integrative or systems biology strategies to achieve the goal of comprehensively understanding the gene-gene network and the gene-environment interaction. More specifically, focuses will be on: (1) unifying phenomics and genomics to identify the genes controlling traits of interest, which can manifest the power of both high-throughput phenotyping and genotyping technologies; (2) unifying bioinformatics, bioengineering and synthetic biology to design new routes for genetic manipulation; (3) combining traditional and modernized breeding techniques such as marker-assisted backcrossing (MAB), marker-assisted recurrent selection (MARC) and genomic selection (GS) for more efficient and precise development of new varieties with desired traits. Such multi-dimensional efforts are expected to solidify the theoretical foundation as well as provide rich genetic resources for the improvement of quality and safety traits in horticultural plants.

## Author Contributions

All authors listed have made a substantial, direct, and intellectual contribution to the work and approved it for publication.

## Conflict of Interest

The authors declare that the research was conducted in the absence of any commercial or financial relationships that could be construed as a potential conflict of interest.

## Publisher's Note

All claims expressed in this article are solely those of the authors and do not necessarily represent those of their affiliated organizations, or those of the publisher, the editors and the reviewers. Any product that may be evaluated in this article, or claim that may be made by its manufacturer, is not guaranteed or endorsed by the publisher.
